# Small nucleolar RNA is potential as a novel player in leukemogenesis and clinical application

**DOI:** 10.1097/BS9.0000000000000091

**Published:** 2021-10-19

**Authors:** Li-Min Lin, Qi Pan, Yu-Meng Sun, Wen-Tao Wang

**Affiliations:** a MOE Key Laboratory of Gene Function and Regulation, State Key Laboratory for Biocontrol, School of Life Sciences, Sun Yat-Sen University, Guangzhou, Guangdong 510275, China

**Keywords:** 2′-O-methylation, Biomarkers, Drug resistance, Leukemia, snoRNA, Targeted therapy

## Abstract

Lack of clarity of the mechanisms that underlie leukemogenesis obstructs the diagnosis, prognosis, and treatment of leukemia. Research has found that small nuclear RNA (snoRNA) plays an essential role in leukemia. These small non-coding RNAs are involved in ribosome biogenesis, including the 2′-O-methylation and pseudouridylation of precursor ribosomal RNA (pre-rRNA), and pre-rRNA splicing. Recently, many snoRNAs were found to be orphans that have no predictable RNA modification targets, but these RNAs have always been found to be located in different subcellular organelles, and they play diverse roles. Using high-throughput technology, snoRNA expression profiles have been revealed in leukemia, and some of the deregulated snoRNAs may regulate the cell cycle, differentiation, proliferation, and apoptosis in leukemic cells and confer drug resistance during leukemia treatment. In this review, we discuss the expression profiles and functions of snoRNAs, particularly orphan snoRNAs, in leukemia. It is possible that the dysregulated snoRNAs are promising diagnosis and prognosis markers for leukemia, which may serve as potential therapeutic targets in leukemia treatment.

## 1 INTRODUCTION

Leukemia is a type of blood cancer caused by the malignant proliferation of hematopoietic cells that can infiltrate the bone marrow, blood, lymph nodes, spleen, and other tissues.^1–4^ Patients with leukemia may suffer severe infections, anemia, and bleeding.^1–4^ According to the 2016 World Health Organization classification guidelines, clinical practice integrates cell morphology, immunophenotype, genetics, and cytogenetics to diagnose and prognose leukemia.^5^ Although several predisposing factors including genetic susceptibility and environmental factors have been identified, the mechanism of leukemogenesis remains unclear, which makes it difficult to diagnose and prognose leukemia in many cases.^1–4^ Further discoveries of the molecular characteristics of leukemia may provide new perspectives on diagnostic and prognostic markers. Recently, due to advances in synthesizing radiotherapy, chemotherapy, allogeneic hematopoietic stem cell transplantation, monoclonal antibodies, CAR-T cells, and single cell and other therapies, great progress has been made in the treatment of leukemia, and the survival rate has significantly improved.^1–4,6–9^ However, the prognosis of older adults remains poor for some subtypes of leukemia, and genotoxic effects and drug resistance continue to hinder treatment.^1–4^ New targeted therapies and treatment options need to be developed.

snoRNAs are a type of small non-coding RNAs that are mainly located in the nucleolus of eukaryotic cells.^10,11^ It has been demonstrated that snoRNAs can be directly transcribed from genes or they may be encoded in the introns of other coding genes.^12^ These molecules are usually complex with diverse sets of proteins to form small nucleolar ribonucleoprotein particles (snoRNPs), which perform multiple functions.^13^ The canonical functions of snoRNAs include ribosomal biogenesis, including the modification and splicing of precursor ribosomal RNA (pre-rRNA),^14,15^ and mediating small nuclear RNA (snRNA) modification.^16^ Some snoRNAs are found to regulate the translation and processing of message RNA (mRNA),^17^ or they function in a manner similar to microRNAs (miRNAs).^18,19^ Dysregulated snoRNAs can affect tumorigenesis and progression by regulating the cell cycle,^20,21^ proliferation,^17,22^ differentiation,^23^ oxidative stress,^24,25^ and apoptosis.^21,22,25^ In recent years, several researchers have also reported the involvement of snoRNAs in the differentiation, proliferation, and apoptosis of leukemic cells and their potential as prognostic biomarkers and therapeutic targets.^26–30^ In this review, we provide an overview of snoRNA function, their expression patterns, and their clinical significance in leukemia. Uncovering the functions of diverse snoRNAs may improve understanding of the underlying biological events in leukemogenesis, ultimately leading to the discovery of novel therapeutic targets and biomarkers and new perspectives for the future design of nucleic acid therapeutics.

## 2 BIOGENESIS AND FUNCTION OF snoRNAs

### 2.1 Biogenesis and location of snoRNAs

**Figure 1:**
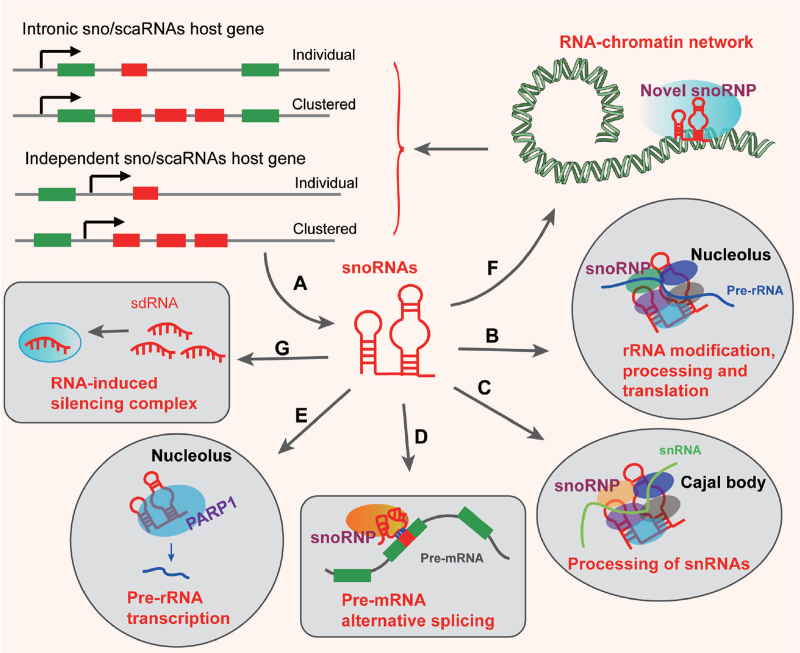
Function of snoRNAs. A. snoRNAs are derived from the snoRNA genes with independent promoters, or they are encoded by intronic snoRNA genes. B. The canonical function of snoRNAs is realized by guiding 2′-O-methylation and pseudouridylation of rRNAs in the nucleolus. C. scaRNAs also guide 2′-O-ribose-methylated nucleotides and pseudouridines on snRNAs in cajal bodies. D. The “orphan” snoRNAs play an important role in alternative splicing. E. snoRNAs bind to PARP1 and stimulate its catalytic activity to promote rDNA transcription. F. snoRNAs are also enriched in chromatin, which suggests a chromatin-associated role. G. snoRNAs can be processed into sno-derived RNA (sdRNA), which has been shown to perform a regulatory function similar to microRNAs. snoRNA = small nuclear RNA.

snoRNAs can be directly transcribed by snoRNA genes with independent promoters, or they may be encoded by intronic snoRNA genes that lack independent promoters (Fig. 1A).^12^ Both types of snoRNAs genes can be distributed individually or in a cluster, and the latter depends on enzymatic processing of a polycistronic precursor RNA to produce a single snoRNA.^12^ Several snoRNAs are transcribed by RNA polymerase II, whereas the transcription of other snoRNAs is driven by the upstream pol III-specific box A and box B.^12^ Based on conserved sequence elements, snoRNAs are classified as C/D box or H/ACA box snoRNAs.

snoRNAs have long been the best-studied class of ncRNAs and have an unglamorous and important role in the protein synthesis machinery. The canonical functions of snoRNA are realized by guiding 2′-O-methylation and pseudouridylation of rRNA in a base-pair dependent manner by C/D box and H/ACA box snoRNAs, respectively (Fig. 1B). However, a subset of snoRNAs called orphan snoRNAs have no predictable RNA modification targets, and therefore largely unknown function. In addition, sporadic reports have indicated that snoRNAs could exert non-canonical functions by mediating alternative splicing, RNA–RNA interactions, processing into small ncRNAs, and forming non-canonical snoRNPs.^31^

In recent years, except for the canonical snoRNAs that are located in the nucleolus and regulate 2′-O-methylation and pseudouridylation during ribosome biogenesis, snoRNAs are found located in many different subcellular organelles and play diverse roles. Several snoRNAs were found in mitochondria to interact with the RNase mitochondrial RNA processing enzyme (MRP), which plays a role in cartilage-hair hypoplasia.^32,33^ These snoRNAs are non-canonical snoRNAs that do not belong to either the C/D box or H/ACA box classes. Small Cajal body (CB)-specific RNAs (scaRNAs) are a subset of canonical snoRNAs that are found in CBs.^16^ It has been found that the location of scaRNAs is dependent on CAB boxes (UGAG) or long UG (GU) dinucleotide repeat elements.^16^ scaRNAs have been demonstrated to function as guide RNAs during the site-specific synthesis of 2′-O-ribose-methylated nucleotides and pseudouridines for RNA polymerase II-transcribed U1, U2, U4, and U5 spliceosomal small nuclear RNAs (snRNAs) (Fig. 1C). Notably, recent evidence of high enrichment of snoRNAs in chromatin-bound fractions open the possibility that they might play a role in chromatin biology.^34–36^ The new tricks for these “old dogs” and the elusive roles of orphan snoRNAs restimulate interest in investigating the diverse functions of snoRNAs, particularly orphan snoRNAs that have no canonical targets.

### 2.2 snoRNAs in ribosome biogenesis

**Figure 2:**
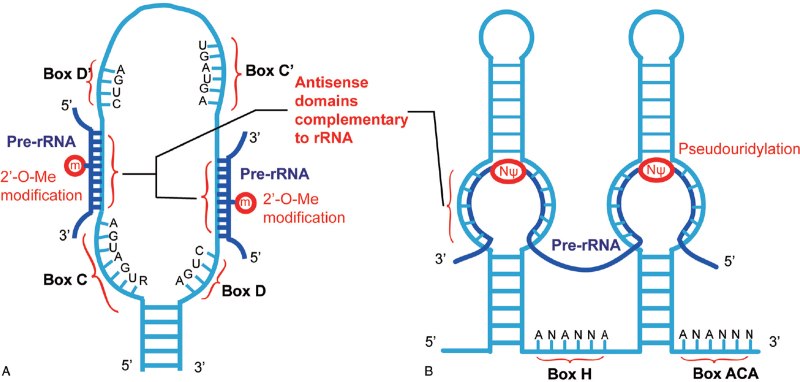
The mechanism of 2′-O-methylated modification and pseudouridylation guided by snoRNAs. The sequence structures of C/D (A) and H/ACA (B) box snoRNAs involved in the 2′-O-methylated modification and pseudouridylation of pre-rRNA, respectively. A long stretch of sequence completely complements the internal sequence of pre-rRNA in which the modification occurs. 2′-O-methylated nucleotides are indicated by m; the pseudouridinylated nucleotide is shown with the sign NΨ. snoRNA = small nuclear RNA.

The canonical roles of snoRNAs are on ribosome biogenesis. A ribosome is a molecular machine for protein synthesis that is responsible for translating the information contained in mRNA into protein. In eukaryotes, the 60S subunit of the ribosome is composed of 28S, 5.8S, and 5S rRNAs and 46 proteins, while the 40S small subunit contains 18S rRNA and 33 proteins.^37^ Ribosome biogenesis in eukaryotes is mainly performed in the nucleolus in a complex process involving the transcription of both ribosomal DNA (rDNA) and ribosomal protein (RP) DNA, the translation of RPs, the modification and processing of pre-rRNA, and the assembly of ribosomal subunits.^15,38^ Numerous lines of evidence have shown that snoRNAs play important roles in the post-transcriptional modification, processing of pre-rRNAs, and ribosome biogenesis. Most snoRNAs are responsible for the 2′-O-methylation of specific nucleotides in pre-RNAs^39^ and the conversion of specific uridines into pseudouridine (Fig. 2).^40^ Some snoRNAs are involved in the processing of pre-rRNAs. For example, U3, U14, snR10, snR30, and MRP/7-2 are necessary for the maturation of yeast 18S rRNA, and U8 plays an important role in the processing of 28S rRNA. Interestingly, there appears to be no consistent positional relationship between snoRNA binding sites and pre-RNA splicing sites.^15^ Thus far, the functional mechanism of snoRNAs involved in pre-RNA processing remains unclear. Whether snoRNAs function as a significant component of the processing complex or if they affect processing by changing the conformation of pre rRNA remains to be verified.^41^

#### 2.2.1 snoRNAs in 2′-O-methylation

The box C/D snoRNA family possesses the conserved nucleotide boxes C and D, which are both conducive to the stability of box C/D snoRNAs.^42^ In the structure of the box C/D snoRNAs, the C and D boxes are respectively located near the 5′ and 3′ ends of the snoRNA.^11^ The two elements are gathered together by their adjacent complementary short sequences, while the middle region of the snoRNA remains loose with the box C′ and box D′ elements, which are 1 to 2 bases away from where box C and box D are located.^11^ Upstream of the D or D′ box exists a long sequence, which can be completely complementary to the internal rRNA sequence.^11^ The two conserved box elements have different functions. The C/C′ boxes are necessary for binding fibrillarin,^43^ while the D or D′ boxes mediate site selection for rRNA 2′-O-methylation by base pairing.^39^ During the 2′-O-methylation mediated by box C/D snoRNAs, the antisense snoRNAs in snoRNPs binds a complementary sequence in pre-RNA to form a double helix, and the nucleotide opposite the fifth nucleotide, which is upstream of the snoRNA D or D′ box, is recognized and modified by fibrillarin (Fig. 2A).^39^ rRNA 2′-O-methylation optimizes the structure of rRNA, leading to the production of ribosomes with high activity and high-fidelity (Fig. 1B).^44^ In addition, rRNA 2′-O-methylation may promote the activity of the translation mediated by the internal ribosome entry site.^45^ These findings suggest that snoRNAs may be involved in some biological events by altering the translation mechanism in cells.

#### 2.2.2 snoRNAs in pseudouridylation

The H/ACA snoRNA family possesses an evolutionarily conserved “hairpin-hinge-hairpin-tail” structure, and the box H and box ACA are respectively located in the single-stranded region and 3′ end of H/ACA snoRNA.^11^ H/ACA snoRNAs mediate site selection of rRNA pseudouridylation (Fig. 2B).^40,46^ The hairpin structure that is adjacent to the H or ACA box forms a pseudouridylation pocket, and this pocket can be complementary to the sequences flanking the rRNA modification site (Fig. 1B).^40,46^ The unpaired uridine (14–16 nt away from the H or ACA box) in the pocket is recognized and converted to pseudouridine by the H/ACA snoRNP protein.^40,46^ Compared to uridine, the pseudouridine in rRNA has three potential functions: (1) the more flexible C–C glycosyl bond may alter the rRNA folding or conformation;^46^ (2) the N-1 proton of pseudouridine can serve as an extra H-bond donor in the intramolecular interaction of rRNA or in rRNA-protein interactions, which may influence rRNA or rRNA-protein conformations;^47,48^ and (3) the N-1 position has a high potential for acyl transfer, which can catalyze the transfer of the growing peptide from the ribosomal P site to the A site and therefore alter the translational activity.^49^ The loss of rRNA pseudouridine may lead to defective small subunits or conformational changes in the peptidyl transferase center (PTC) of large ribosome subunits, therefore impairing the translation activity of ribosomes.^50^ Additionally, the pseudouridine in rRNA was found to have the potential to stabilize its adjacent nucleotides and was therefore suggested to influence the fidelity of the ribosome by stabilizing the key nucleotide in PTC.^50^ However, it appears that not all pseudouridines in rRNA are indispensable.^46,50^ Highly conserved pseudouridines may function significantly, while others may have subtle or even no effect. In addition, the loss of more than one snoRNAs was found to improve the subtle effects caused by a single snoRNA, suggesting that synergy may exist among cellular snoRNAs.^50^ The functional mechanism of snoRNA-mediated pseudouridylation is complicated, and analysis of H/ACA snoRNA expression profiles based on high throughput technology may provide new perspectives.

#### 2.2.3 snoRNAs with other functions

Recent studies have shown that snoRNAs possess additional functions. MRP RNA is a non-canonical snoRNA that is an important component of ribonuclease mitochondrial RNA processing (RNase MRP) that plays an important role in pre-rRNA processing and cell-cycle regulation.^14^ It has been reported that MRP RNA can also serve as a component of RNase P to regulate the processing of pre-tRNA.^11^ RNase MRP and RNase P are closely related in both evolution and structure; however, their substrate specificity and substrate recognition mechanism are quite different, leading to distinct functions.^14^ In addition, scaRNAs contain a C/D box and/or a H/ACA box, and they serve as regulatory molecules that mediate post-transcriptional modification of spliceosomal small nuclear RNAs, including 2′-O-methylation and pseudouridylation (Fig. 1C).^51^ These modifications are essential to form an active spliceosome, and influence the subsequent splicing of pre-mRNA.^52^ Recently, approximately half of the human snoRNAs were termed “orphans” because they have no sequence complementarity and lack known targets, which suggests additional functions for this class of RNAs. For instance, the “orphan” neuron-specific SNORD115 plays an important role in alternative splicing.^53,54^ SNORD115 binds to the dsRNA structure of serotonin receptor 2C pre-mRNA and provides the inclusion of exon Vb into the mRNA, generating a normal receptor with high sensitivity to serotonin (Fig. 1D).^53,54^ Lykke-Andersen et al reported that snoRD86 acts in cis as a sensor and controls the alternative splicing of its host transcript Nucleolar Protein 56 (NOP56) via a structural “switching” mechanism.^55^ SNORD88C has also been demonstrated to have base complementarity to an intronic region of fibroblast growth factor receptor 3 (FGFR3), and it increases exon inclusion in the FGFR3 gene transcript.^56^ Interestingly, snoRNAs have also been reported to bind to poly ADP-ribose polymerase 1 (PARP1), a ubiquitously expressed nuclear enzyme that plays key roles in DNA repair and gene regulation.^57^ However, snoRNAs stimulate PARP1 catalytic activity in the nucleolus independent of DNA damage. Activated PARP1 ADP-ribosylates DDX21, an RNA helicase, and then localizes to nucleoli and promotes rDNA transcription (Fig. 1E).^57^ The selectivity activation modes of special snoRNAs may suggest that snoRNAs can change traditional regulatory outcomes. Of note, several snoRNAs are enriched in chromatin and are suggested to play a role in chromatin biology.^58^ Drosophila decondensation factor 31 (Df31) mediates the link between snoRNAs and chromatin and regulates an RNA-chromatin network, resulting in the establishment of open chromatin domains and RNA transcription (Fig. 1F).^58^

Recent studies have revealed that a large proportion of snoRNAs are further processed into smaller molecules.^18,19,59^ The function of these small molecules is mostly unknown except for a subset of them that has been shown to perform a regulatory function like microRNAs (Fig. 1G).^18,19,59^ Interestingly, sunny Sharma et al demonstrate that a novel role for box C/D snoRNAs can guide 18S rRNA acetylation and aid early pre-rRNA processing in yeast.^60^ Further research of the relationship between snoRNAs and their processed smaller molecules and diverse rRNA modifications may contribute to the understanding of the unknown functions of snoRNAs.

## 3 EXPRESSION AND FUNCTION OF SNORNAS IN LEUKEMIA

According to disease progression and cell lineage, leukemia can be divided into several subtypes: chronic lymphocytic leukemia (CLL), chronic myeloid leukemia, acute lymphocytic leukemia (ALL), and acute myeloid leukemia (AML).^61,62^ In recent years, snoRNAs have been found to have different expression profiles in the different leukemia subtypes, and they regulate leukemogenesis and progression through diverse regulation modes.

### 3.1 snoRNAs in AML

**Figure 3:**
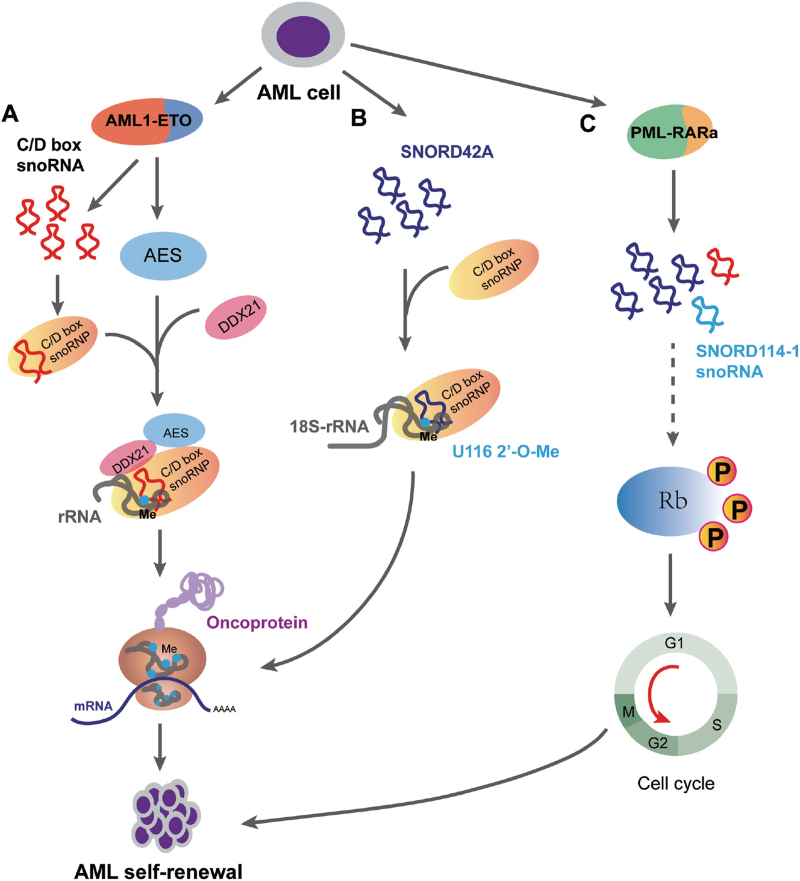
Regulation of C/D box snoRNAs in AML self-renewal. A. The schematic representation describes the regulation of C/D box snoRNA in AML-ETO AML. AML1-ETO induces AES and snoRNAs. AES facilitates maintenance of C/D box snoRNAs and ribosomal RNA 2′-O-methylation via binding to DDX21, resulting in the promotion of oncoprotein synthesis and impairment of translation fidelity. AES: amino-terminal enhancer of split; DDX21: RNA helicase. B. Highly expressed SNORD42A directs 2′-O-methylation at uridine 116 in 18S ribosomal RNA (rRNA), and specifically increases the translation of ribosomal oncoproteins. C. Downstream of PML–RAR alpha, SNORD114-1 promotes cell growth through cell cycle modulation by mediating the phosphorylation of the Rb to promote the G0/G1 to S phase transition. Rb = retinoblastoma protein, snoRNA = small nuclear RNA.

AML is a malignant disorder of hemopoietic stem cells characterized by clonal expansion of abnormally differentiated myeloid lineage blasts.^63^ A few snoRNAs are regulated in a lineage- and development-specific manner and play a role in the biogenesis and progression of AML (Fig. 3). In t(8;21) AML, the expression of a subset of C/D box snoRNAs was stimulated by the fusion gene and subsequently promoted the self-renewal of AML cells (Fig. 3A).^26^ The fusion protein AML1-ETO induces the expression of the amino-terminal enhancer of split (AES), which subsequently up-regulates the expression of C/D box snoRNA and promotes the formation of snoRNP by interacting with the RNA helicase DDX21.^26^ Similarly, other AML-related oncogenes, including MYC and MLL-AF9, can also up-regulate snoRNA expression, but this process does not depend on AES.^26^ These data indicate that the regulation of AML-related oncogenes by snoRNA is complicated. Although the functional mechanism by which C/D box snoRNPs promote the proliferation of AML cells is unclear, research has shown that a decrease in the level of 2′-O-methylation is accompanied by down-regulation of C/D box snoRNAs.^26^ It has been reported that C/D box snoRNAs mediate the 2′-O-methylation of pre-rRNA, and this modification may influence the translational activity and fidelity of ribosomes.^39,44,45^ Additionally, acceleration of protein synthesis was suggested to be necessary for the proliferation of cancer cells.^38^ Therefore, it is reasonable that C/D box snoRNAs may regulate AML by changing the translational activity of ribosomes through mediating the 2′-O-methylation of pre-rRNA. Interestingly, a subset of snoRNAs related to ribosome biogenesis was found to be dysregulated in AML.^27^ For instance, SNORD42A was reported to act as a C/D box snoRNA to mediate the 2′-O-methylation of 18S-U116 in the 40S subunit, which may change the translation preference of the ribosome by affecting its 3D structure, thereby promoting cell proliferation (Fig. 3B).^29^

In addition to the canonical functions of pre-rRNA modification, recent studies have shown that snoRNAs possess additional functions in AML progression. The DLK1-DIO3 locus, which is located in imprinted regions, was found to be related to leukemogenesis and progression. This locus encodes a number of ncRNAs, including 41 snoRNAs, 11 lncRNAs, and 53 miRNAs,^27^ and the snoRNAs have been reported to be expressed in a lineage- and development-specific manner during hematopoiesis.^27^ For example, the expression of these snoRNAs was highest in CD34^+^ cells, and it rapidly decreased throughout differentiation, remaining at a low level in mature neutrophils.^27^ In the DLK1-DIO3 locus, a variety of snoRNAs were down-regulated in AML and related to both ribosome biogenesis and the mRNA splicing complex, while SNORD112-114 was up-regulated in promyelocytic leukemia.^20^ Among these snoRNAs, SNORD114-1 could regulate the cell cycle transition (from G0/G1 to S phase) mediated by the Rb/p16 pathway to promote the growth of AML cells (Fig. 3C).^20^ The snoRNAs encoded by the DLK1-DIO3 locus might serve as regulatory molecules in several biological events involved in AML, including the development of the hematopoietic system, ribosome translation, mRNA processing, and cell cycle transition.^20,27^ Another study has shown that epigenetic silencing of the DLK1-DIO3 locus inhibits the pluripotency of induced pluripotent stem cells (iPSCs).^64^ The roles of the intronic snoRNAs encoded in the DLK1-DIO3 locus in the pluripotency of stem cells remain to be studied.^64^

Of note, genes related to ribosome biogenesis have been found to be core targets of the oncogene MYC, and some of these encode snoRNAs.^65^ MYC directly binds to promoter sequences (CACGTG) of snoRNA genes to enhance their transcription. For example, snoRNAs encoded by U-snoRNA host genes (Uhg) could be directly stimulated by MYC.^65^ Moreover, MYC could regulate the expression of target snoRNAs in an indirect manner, likely by activating their host genes or by affecting their stability.^65,66^ Mutations in target snoRNAs in sites associated with 2′-O-methylation weaken MYC's ability to promote nuclear growth, suggesting that these snoRNAs are important downstream effectors of MYC function.^65^ These findings provide the novel perspective that snoRNAs can be regulated by oncogenes and subsequently affect the efficiency of protein synthesis, leading to effects on leukemogenesis and progression. Although the specific targets of snoRNAs in AML appear to be largely uncommon, the contribution of differentially expressed snoRNAs on the regulation of hematological malignancies suggests an exciting new area of investigation.

### 3.2 snoRNAs in ALL

ALL involves the malignant proliferation of lymphoid cells blocked at an early stage of differentiation.^2^ In ERG-related leukemia, a subtype of B cell precursor acute lymphoblastic leukemia (BCP-ALL), several snoRNAs in the Prader-Willi locus were found to be specifically upregulated, including SNORD109A, SNORD64, SNORD107, and 12 snoRNAs in the SNORD116 cluster (SNORD116-11, -14, -15, -16, -17, -18, -20, -21, -22, -23, -24, and -27) (Table 1).^67^ Similarly, SNORD116-18 was also upregulated in CLL patients and found to be associated with shorter progression-free survival (PFS).^68^ Thus far, most of the snoRNAs in the Prader-Willi locus except for SNORD115 are orphan snoRNAs with no known target or function.^69^ SNORD115 was reported to regulate the alternative splicing of serotonin receptor 2C pre-mRNA, and its loss may contribute to Prader-Willi syndrome.^53,54^ The hypothesis that some orphan snoRNAs in the Prader-Willi locus function in the same manner as SNORD115 remains to be verified.

**Table 1:** Summary of the roles of snoRNAs in leukemia.

snoRNAs	Subtype	Expression	Function	Reference
SNORD34, SNORD35A, SNORD43	AML1-ETO AML	Up	rRNA methylation, protein synthesis clonogenic growth	^28^
SNORD14D	AML1-ETO AML	Up	Clonogenic growth, protein synthesis without affecting rRNA methylation	^28^
SNORD14D, SNORD35A	Biphenotypic B-myelomonocytic leukemia	Up	Colony formation	^28^
SNORD114–1, SNORD112, SNORD113–6, SNORD113–7, SNORD113–8, SNORD113–9	acute promyelocytic leukemia (APL)	Up	Cell growth through cell cycle modulation	^20^
SNORD109A, SNORD64, SNORD107 and 12 snoRNAs in the SNORD116 cluster (SNORD116-11, -14, -15, -16, -17, -18, -20, -21, -22, -23, -24, -27)	B cell precursor acute lymphoblastic leukemia (BCP ALL)	Up	Unknown	^66,67^
SNORD116-18, SNORA74A	CLL	Up	Associated with shorter PFS	^67^
SNORD56, SNORD1A, SNORA70F	CLL	Down	Associated with shorter PFS	^67^
SNORA31, -6, -62, and 71C	CLL	Down	Guide target rRNAs pseudouridylation	^67^
SNORA12, -22, -27, -56, -64, -69, -70, -74A, -80, -84; SNORD1A, -1B, -8, 18A, -30, -32A, -34, -105B, -110; SCARNA8	IGHV-M CLL	Up	Associated with shorter TFS, cell proliferation	^24^
U50, U50′	B-cell lymphoma	Normal	Cell growth	^74,75,76^
ACA11	t(4;14)-positive multiple myeloma	Up	Oxidative stress, doxorubicin resistance, cell growth	^22,23,26^

Growth Arrest Specific 5(GAS5) is a multi-small-nucleolar-RNA host gene that can be transcribed into long non-coding RNA (lncRNA GAS5) and encode snoRNA.^70^ GAS5 plays an essential role in growth arrest and apoptosis.^70,71^ Overexpression of GAS5 leads to an increase in cell apoptosis and a cell cycle arrest in both leukemic and normal T lineage cells.^70^ In Friend leukemia and breast cancer, GAS5 was reported to be downregulated, which leads to apoptosis inhibition and aberrant proliferation.^71,72^ Similarly, RNU44, a snoRNA encoded by GAS5, was normally upregulated to arrest cell growth under stress, and its lower expression is associated with poor prognosis in breast cancer.^21^ The relationship between the functional mechanism of GAS5 and that of RNU44 remains unknown. However, sequence analysis reveals that only intronic snoRNAs are highly conserved in GAS5, suggesting that snoRNAs significantly contribute to GAS5 function.^21^ As the aberrant proliferation of T lineage cells can lead to ALL,^2^ aberrant growth arrest in T lineage cells by GAS5, or perhaps by the intronic snoRNA, may significantly affect ALL development.^70^ Moreover, GAS5 is involved in chromosomal translocation in lymphoma, for example, the GAS5 gene fused to BCL6 was found in B-cell lymphoma.^73^ Whether snoRNAs co-transcribe with the GAS5 host gene and have functional effects in lymphoid leukemia requires further research.

### 3.3 snoRNAs in CLL

CLL is a lymphoproliferative disorder that is characterized by the expansion of neoplastic B lymphocytes in the peripheral blood, secondary lymphoid tissues, and bone marrow.^3^ An analysis of the sno/scaRNA expression profile in CLL demonstrated that SNORA31, SNORA6, SNORA62, and SNORA71C are all down-regulated (Table 1).^67^ These snoRNAs are canonical H/ACA snoRNAs, which mediate the site-specific pseudouridineization of rRNA.^67^ It is not clear whether these snoRNAs have pathological effects; however, it is worth noting that their host genes play roles in tumor-related signal transduction.^67^ For example, TPT1, the host gene of SNORA31, can directly or indirectly regulate the expression of the p53 gene and consequently influence cell growth, proliferation, tumor reversal, and reprogramming of both embryonic stem cells and tumor cells.^74^ It has been reported that upregulated expression of SNORD116-18, SNORA70F, SNORA74A, SNORD56, and SNORD1A is relevant to shorter PFS in CLL patients.^67^ Although the functions of these snoRNAs remain unclear, they were suggested to be involved in the regulation of alternative splicing mRNAs.^53,54^ The snoRNA expression profiles of additional CLL cases were analyzed in another study reporting that the overexpression of 20 snoRNAs was associated with the short treatment-free survival (TFS) of IGHV-mutated (IGHV-M) patients, and that most of the 20 snoRNAs could be functionally relevant in CLL cell proliferation.^75^ Furthermore, after CLL cells were induced to proliferate, 7 snoRNAs continued to be up-regulated with proliferation, while 2 snoRNAs (SNORA80 and SNORD1A) were down-regulated.^75^ It is evident that snoRNAs are subject to complex regulation during the proliferation of CLL cells. Some snoRNAs may be deregulated during proliferation, whereas others may continue to be up-regulated, and the latter likely have important regulatory functions.

### 3.4 snoRNAs in other hematologic neoplasms

snoRNAs also have functional effects in other hematologic neoplasms. The intronic U50 snoRNA gene is located at chromosomal breakpoint t(3;6)(q27;q15) in human B-cell lymphoma, and the encoded U50/U50’ snoRNA acts as C/D box snoRNA to mediate site-specific 2′-O-methylation in 28S rRNA.^76^ 2′-O-methylation has been considered a promising functional mechanism of U50/U50′ snoRNA in lymphomagenesis.^76^ Although the role of U50 snoRNA is unclear, it has been suggested that U50 snoRNA is a candidate for the 6q tumor suppressor gene in other human cancers.^77,78^ For example, snoRNA U50 usually undergoes a copy loss, a 2-bp deletion, and downregulation in both breast and prostate cancer, and re-expression of snoRNA U50 was found to inhibit the colony-forming ability of breast cancer cells, suggesting that snoRNA U50 has the potential to suppress tumorigenesis.^77,78^ An orphan box H/ACA snoRNA, ACA11, was found to be highly expressed in t(4;14)-positive multiple myeloma (MM), and this snoRNA suppressed oxidative stress, provided resistance to chemotherapy, and increased the proliferation of MM cells.^24^ Unlike canonical H/ACA snoRNA, ACA11 appears to be involved in an RNA processing complex,^24^ suggesting that it may regulate cellular RNA processing. Further research showed that ACA11 regulates the ROS level by inhibiting the expression of nuclear factor (erythroid-derived 2)-related factor 2 (NRF2), and it stimulated ribosome biogenesis and protein synthesis in a ROS-dependent manner to promote proliferation.^79,80^ Although these discoveries have revealed a partial mechanism of ACA11, the targets of this orphan snoRNA remains unknown. A study of the sno/scaRNA profile in MM has revealed the specific dysregulated snoRNAs and their potential function.^81^ Unveiling the direct targets of these snoRNAs may provide new perspectives on their functional mechanism.

## 4 EXPRESSION PATTERN OF LEUKEMIA-RELATED SNORNAS AND THEIR CLINICAL SIGNIFICANCE

### 4.1 Leukemia-related snoRNAs in drug resistance

With the development of chemotherapy and molecular targeting drugs, the treatment of leukemia has improved, leading to an increased survival rate.^1–4^ However, drug resistance remains a limiting factor in leukemia treatment.^1–4^ The discoveries of snoRNAs have provided new perspectives for dealing with drug resistance in leukemia. GAS5, a multi-small-nucleolar-RNA host gene, regulates apoptosis and the growth arrest of T cells,^70^ and its downregulation promoted Friend leukemia.^72^ Interestingly, SNORD44, an intronic snoRNA in GAS5, was reported to regulate apoptosis and growth arrest in breast cancer.^21^ Because apoptosis failure is tightly associated with drug resistance in cancer,^21^ GAS5 and SNORD44 may play essential roles in drug resistance. This hypothesis was partially confirmed by the report that down-regulation of GAS5 rescued leukemic T cells from rapamycin, an mTOR antagonist.^82^ As SNORD44 is highly conserved in GAS5, it has been suggested to be a significant functional component of the GAS5 gene,^21^ which is consistent with a study reporting that it shares a similar function with GAS5.^70^ Together, these findings suggest that SNORD44 may be involved in rapamycin drug resistance in leukemia. Although relevant research in leukemia is limited, the evidence that snoRNAs function in drug resistance has been reported in other human cancers.^24^ For example, the expression of the H/ACA box snoRNA ACA11 in MM.1S cells reduced the level of ROS and increased resistance to doxorubicin, suggesting that ACA11 rescues cells from cytotoxic chemotherapy (Table 1).^24^ Indeed, snoRNAs have become promising targets for treatment in drug-resistant leukemia.

### 4.2 Leukemia-related snoRNAs in targeted therapy

snoRNAs may affect the biogenesis and progression of leukemia by regulating cell cycle,^20,21^ proliferation,^17,75,81^ differentiation,^23^ and apoptosis,^21,22,25^ suggesting that they are potential targets for leukemia treatment (Table 1). Some snoRNAs have been demonstrated to be upregulated in AML and promote this disease by altering ribosome activity. For example, SNORD42A was reported to mediate the 2′-O-methylation of 18S-U116 rRNA in the 40S subunit, which might alter the translation preference of the ribosome, consequently promoting proliferation.^29^ A portion of snoRNAs regulate the cell cycle of cancer cells. It was reported that SNORD114-1 regulates the cell cycle transition mediated by the Rb/p16 pathway in AML.^20^ Some studies on the sno/scaRNA profile have revealed dysregulated snoRNAs in different subtypes of leukemia, including SNORD109A, SNORD64, SNORD107, SNORD116-18, SNORA31, SNORA6, SNORA62, SNORA71C, SNORA70F, SNORA74A, SNORD56, and SNORD1A.^67,68^ Among the dysregulated snoRNAs, SNORD116-18, SNORA70F, SNORA74A, SNORD56, and SNORD1A have been associated with shorter PFS for CLL patients.^67^ These snoRNAs are promising targets for leukemia therapy. On one hand, treatments should be administered to control the level of dysregulated snoRNAs in leukemia. We can improve the level of snoRNAs that suppress leukemia by transporting exogenous snoRNAs into leukemic cells, whereas the expression of snoRNAs that promote leukemia should be inhibited. On the other hand, competing for the downstream targets of snoRNAs may be a good strategy in targeted therapy whereby snoRNA function will be blocked.

### 4.3 Leukemia-related snoRNAs as diagnostic/prognostic markers

The sno/scaRNA profile of leukemia has revealed that some snoRNAs are specifically upregulated or downregulated in different subtypes, suggesting that these snoRNAs are promising diagnosis and prognosis biomarkers. The sno/scaRNA expression profiles of CLL were suggested to be heterogeneous in different subgroups,^68,75^ and expression of SNORA74A and SNORD116-18 could indicate two CLL groups with different PFS (Table 1).^68^ Similarly, there were significant differences between the snoRNA expression patterns in these leukemia subtypes, including AML, pre-B-ALL, and T-ALL.^28^ These findings indicate that the characteristics of snoRNA expression could be potential biomarkers for diagnosis and prognosis in leukemia, which may contribute to more effective treatment.

## 5 STRATEGIES USED TO STUDY THE ROLES AND MECHANISMS OF SNORNAS IN LEUKEMIA

snoRNAs are a class of metabolically stable RNAs, 60–300 nucleotide in length, that are excised from the intron regions of pre-mRNAs and located in the nucleolus.^10,11^ During the last decade, with the rapid development of biotechnology, snoRNAs have been found to have many novel, unexpected cellular functions and have been associated with leukemogenesis.^26–30^ Although several studies have reported the expression profiles and roles of snoRNAs in leukemia, the strategies used to uncover the comprehensive functions of snoRNAs on leukemogenesis are still defective. Here, we try to discuss and summarize the potential technologies used to study the roles and mechanisms of snoRNAs in leukemia. Firstly, genome-wide analysis with different omics techniques including GeneChip microarray on snoRNAs, high-throughput quantitative PCR of snoRNAs, and next-generation sequencing approaches, were applied to identify and quantify the global dysregulated snoRNAs in different subtypes of leukemia.^20,27^ Interestingly, Warner et al modified the cDNA library generation, which added an oligonucleotide adaptor to the 39-end of RNA before reverse transcription.^27^ Selecting the RNA species between 17 and 200 nucleotides, which includes snoRNAs and other sncRNAs and excludes most messenger RNA (mRNA) molecules, they obtained both annotated and novel snoRNAs using 2 complementary bioinformatics approaches.^27^ Following the genome-wide analysis, a real-time PCR assay with specific primers or northern blot with probes was always used to verify the dysregulated snoRNAs. Secondly, the majority of snoRNAs function as guide RNAs that target directly the rRNAs by base-pairing in the post-transcriptional synthesis of 2′-O-methylated nucleotides and pseudouridines of rRNAs.^39^ There are many databases that were used to predict and search for the possible targeted rRNAs, such as, snOPY,^83^ snoRNABase,^84^ and snoSeeker.^85^ As for the unknown site of methylate, the 2′-O-methylated (Nm)-seq or Ψ-seq were used to identify.^86,87^ To verify the target of classical snoRNAs, a site-specific primer is designed to quantify the ribose methylation of pre-rRNA. For example, for detection of 2′-O-methylation on 18 s rRNA cytidine^1703^, a series of specific primers were designed to be used in the PCR procedure under high dNTP (1 mM) and low dNTP (10 μM) concentration, and the PCR products were loaded and separated on 2% agarose gels.^26^ SiRNA, shRNA, and CRISPR/CAS9 were generally used to knock down or out the snoRNA and then the diverse functions in leukemia were detected, such as protein translation, cell differentiation, and cell growth.^20,21^ Of note, many of new-found snoRNAs called orphan snoRNAs have no predictable RNA modification targets, and therefore largely unknown function. However, orphan snoRNAs are unlikely to act alone, and always bind novel partners through their non-canonical structure,^88^ mass spectrometry was generally applied to identify unique protein shared with pull-down of snoRNA and followed by RNA immunoprecipitation.^57,89^ Since snoRNA has been found to become one of the important factor in leukemogenesis, more optimal strategies were needed to reveal the comprehensive functions and pathogenesis mechanisms of snoRNAs in the initiation and progression of leukemia in the future.

## 6 CONCLUSION

snoRNAs are involved in diverse cellular processes, including modification and splicing of pre-rRNA,^14,15^ snRNA modification,^16^ and the translation and processing of mRNA.^17^ The 2′-O-methylation and pseudouridylation mediated by snoRNAs likely alter the activity of ribosomes, therefore promoting the biogenesis and progression of leukemia. In addition, because miRNAs have been reported to have a significant function in human cancer,^90^ it is likely that snoRNAs, which can be processed into miRNAs, may also play an essential role in leukemia. Undoubtedly, unveiling the novel functions of snoRNAs contributes to a more comprehensive understanding of leukemia.

Of note, in recent years, several snoRNAs were found to be located in many different subcellular organelles, and many of them are orphan molecules that may function by forming non-canonical snoRNPs. The elusive roles of orphan snoRNAs restimulate interest in investigating the diverse functions and regulatory mechanisms of snoRNAs, particularly their roles in leukemia. Furthermore, snoRNA expression profiles based on high-throughput technology have revealed some dysregulated snoRNAs in leukemia, which provide the novel perspective that snoRNAs are promising biomarkers for diagnosis and prognosis and that they can serve as targets for treatment, particularly in drug resistance. Further research on the direct targets of the dysregulated snoRNAs may decrypt their functional mechanism. As for targeted therapy based on snoRNAs, there may be two potential strategies. One strategy is controlling the level of the dysregulated snoRNAs in leukemia, and the other focuses on competing for downstream targets of snoRNAs. However, there may be a long time before snoRNA-based therapy is used in clinical treatment. More comprehensive and more targeted research will further reveal the clinical significance of snoRNAs.

## References

[1] Short NJ Rytting ME Cortes JE. Acute myeloid leukaemia. *Lancet* 2018;392 (10147):593–606. 30078459 10.1016/S0140-6736(18)31041-9 PMC10230947

[2] Malard F Mohty M. Acute lymphoblastic leukaemia. *Lancet* 2020;395 (10230):1146–1162. 32247396 10.1016/S0140-6736(19)33018-1

[3] Bosch F Dalla-Favera R. Chronic lymphocytic leukaemia: from genetics to treatment. *Nat Rev Clin Oncol* 2019;16 (11):684–701. 31278397 10.1038/s41571-019-0239-8

[4] Apperley JF. Chronic myeloid leukaemia. *Lancet* 2015;385 (9976):1447–1459. 25484026 10.1016/S0140-6736(13)62120-0

[5] Arber DA Orazi A Hasserjian R The 2016 revision to the World Health Organization classification of myeloid neoplasms and acute leukemia. *Blood* 2016;127 (20):2391–2405. 27069254 10.1182/blood-2016-03-643544

[6] Huang D Sun G Hao X ANGPTL2-containing small extracellular vesicles from vascular endothelial cells accelerate leukemia progression. *J Clin Invest* 2021;131 (1):e138986. 10.1172/JCI138986 PMC7773400 33108353

[7] Zhou F Li X Wang W Tracing haematopoietic stem cell formation at single-cell resolution. *Nature* 2016;533 (7604):487–492. 27225119 10.1038/nature17997

[8] Jin Z Luo Q Lu S Oligoclonal expansion of TCR Vdelta T cells may be a potential immune biomarker for clinical outcome of acute myeloid leukemia. *J Hematol Oncol* 2016;9 (1):126. 27863523 10.1186/s13045-016-0353-3 PMC5116135

[9] Kasakovski D Xu L Li Y. T cell senescence and CAR-T cell exhaustion in hematological malignancies. *J Hematol Oncol* 2018;11 (1):91. 29973238 10.1186/s13045-018-0629-x PMC6032767

[10] Weinberg RA Penman S. Small molecular weight monodisperse nuclear RNA. *J Mol Biol* 1968;38 (3):289–304. 5718554 10.1016/0022-2836(68)90387-2

[11] Tollervey D Kiss T. Function and synthesis of small nucleolar RNAs. *Curr Opin Cell Biol* 1997;9 (3):337–342. 9159079 10.1016/s0955-0674(97)80005-1

[12] Dieci G Preti M Montanini B. Eukaryotic snoRNAs: a paradigm for gene expression flexibility. *Genomics* 2009;94 (2):83–88. 19446021 10.1016/j.ygeno.2009.05.002

[13] Smith CM Steitz JA. Sno storm in the nucleolus: new roles for myriad small RNPs. *Cell* 1997;89 (5):669–672. 9182752 10.1016/s0092-8674(00)80247-0

[14] Lan P Zhou B Tan M Structural insight into precursor ribosomal RNA processing by ribonuclease MRP. *Science* 2020;369 (6504):656–663. 32586950 10.1126/science.abc0149

[15] Eichler DC Craig N. Processing of eukaryotic ribosomal RNA. *Prog Nucleic Acid Res Mol Biol* 1994;49:197–239. 7863007 10.1016/s0079-6603(08)60051-3

[16] Richard P Darzacq X Bertrand E Jády BE Verheggen C Kiss T. A common sequence motif determines the Cajal body-specific localization of box H/ACA scaRNAs. *EMBO J* 2003;22 (16):4283–4293. 12912925 10.1093/emboj/cdg394 PMC175784

[17] Huang C Shi J Guo Y A snoRNA modulates mRNA 3′ end processing and regulates the expression of a subset of mRNAs. *Nucleic Acids Res* 2017;45 (15):8647–8660. 28911119 10.1093/nar/gkx651 PMC5587809

[18] Scott MS Ono M. From snoRNA to miRNA: dual function regulatory non-coding RNAs. *Biochimie* 2011;93 (11):1987–1992. 21664409 10.1016/j.biochi.2011.05.026 PMC3476530

[19] Ender C Krek A Friedländer MR A human snoRNA with microRNA-like functions. *Mol Cell* 2008;32 (4):519–528. 19026782 10.1016/j.molcel.2008.10.017

[20] Valleron W Laprevotte E Gautier EF Specific small nucleolar RNA expression profiles in acute leukemia. *Leukemia* 2012;26 (9):2052–2060. 22522792 10.1038/leu.2012.111

[21] Gee HE Buffa FM Camps C The small-nucleolar RNAs commonly used for microRNA normalisation correlate with tumour pathology and prognosis. *Br J Cancer* 2011;104 (7):1168–1177. 21407217 10.1038/sj.bjc.6606076 PMC3068486

[22] Mei YP Liao JP Shen J Small nucleolar RNA 42 acts as an oncogene in lung tumorigenesis. *Oncogene* 2012;31 (22):2794–2804. 21986946 10.1038/onc.2011.449 PMC4966663

[23] Zhang Y Xu C Gu D H/ACA box small nucleolar RNA 7A promotes the self-renewal of human umbilical cord mesenchymal stem cells. *Stem Cells* 2017;35 (1):222–235. 27573912 10.1002/stem.2490

[24] Chu L Su MY Maggi LB Multiple myeloma-associated chromosomal translocation activates orphan snoRNA ACA11 to suppress oxidative stress. *J Clin Invest* 2012;122 (8):2793–2806. 22751105 10.1172/JCI63051 PMC3408744

[25] Michel CI Holley CL Scruggs BS Small nucleolar RNAs U32a, U33, and U35a are critical mediators of metabolic stress. *Cell Metab* 2011;14 (1):33–44. 21723502 10.1016/j.cmet.2011.04.009 PMC3138526

[26] Zhou F Liu Y Rohde C AML1-ETO requires enhanced C/D box snoRNA/RNP formation to induce self-renewal and leukaemia. *Nat Cell Biol* 2017;19 (7):844–855. 28650479 10.1038/ncb3563

[27] Warner WA Spencer DH Trissal M Expression profiling of snoRNAs in normal hematopoiesis and AML. *Blood Adv* 2018;2 (2):151–163. 29365324 10.1182/bloodadvances.2017006668 PMC5787869

[28] Teittinen KJ Laiho A Uusimäki A Pursiheimo JP Gyenesei A Lohi O. Expression of small nucleolar RNAs in leukemic cells. *Cell Oncol (Dordr)* 2013;36 (1):55–63. 23229394 10.1007/s13402-012-0113-5 PMC13012689

[29] Pauli C Liu Y Rohde C Site-specific methylation of 18S ribosomal RNA by SNORD42A is required for acute myeloid leukemia cell proliferation. *Blood* 2020;135 (23):2059–2070. 32097467 10.1182/blood.2019004121

[30] Dixon-McIver A East P Mein CA Distinctive patterns of microRNA expression associated with karyotype in acute myeloid leukaemia. *PLoS One* 2008;3 (5):e2141. 18478077 10.1371/journal.pone.0002141 PMC2373886

[31] Falaleeva M Welden JR Duncan MJ Stamm S. C/D-box snoRNAs form methylating and non-methylating ribonucleoprotein complexes: old dogs show new tricks. *Bioessays* 2017;39 (6). 10.1002/bies.201600264. 10.1002/bies.201600264 PMC5586538 28505386

[32] Steinbusch MMF Caron MMJ Surtel DAM The antiviral protein viperin regulates chondrogenic differentiation via CXCL10 protein secretion. *J Biol Chem* 2019;294 (13):5121–5136. 30718282 10.1074/jbc.RA119.007356 PMC6442052

[33] Chen XS Penny D Collins LJ. Characterization of RNase MRP RNA and novel snoRNAs from *Giardia intestinalis* and *Trichomonas vaginalis*. *BMC Genomics* 2011;12:550. 22053856 10.1186/1471-2164-12-550 PMC3228867

[34] Li X Zhou B Chen L Gou LT Li H Fu XD. GRID-seq reveals the global RNA-chromatin interactome. *Nat Biotechnol* 2017;35 (10):940–950. 28922346 10.1038/nbt.3968 PMC5953555

[35] Zhou B Li X Luo D Lim DH Zhou Y Fu XD. GRID-seq for comprehensive analysis of global RNA-chromatin interactions. *Nat Protoc* 2019;14 (7):2036–2068. 31175345 10.1038/s41596-019-0172-4 PMC7721247

[36] Yin Y Lu JY Zhang X U1 snRNP regulates chromatin retention of noncoding RNAs. *Nature* 2020;580 (7801):147–150. 32238924 10.1038/s41586-020-2105-3 PMC12018070

[37] Ben-Shem A Garreau de Loubresse N Melnikov S Jenner L Yusupova G Yusupov M. The structure of the eukaryotic ribosome at 3.0Å resolution. *Science* 2011;334 (6062):1524. 22096102 10.1126/science.1212642

[38] Pelletier J Thomas G Volarević S. Ribosome biogenesis in cancer: new players and therapeutic avenues. *Nat Rev Cancer* 2018;18 (1):51–63. 29192214 10.1038/nrc.2017.104

[39] Kiss-László Z Henry Y Bachellerie J-P Caizergues-Ferrer M Kiss T. Site-specific ribose methylation of preribosomal RNA: a novel function for small nucleolar RNAs. *Cell* 1996;85 (7):1077–1088. 8674114 10.1016/s0092-8674(00)81308-2

[40] Ganot P Bortolin M-L Kiss T. Site-specific pseudouridine formation in preribosomal RNA is guided by small nucleolar RNAs. *Cell* 1997;89 (5):799–809. 9182768 10.1016/s0092-8674(00)80263-9

[41] Kiss T. Small nucleolar RNAs: an abundant group of noncoding RNAs with diverse cellular functions. *Cell* 2002;109 (2):145–148. 12007400 10.1016/s0092-8674(02)00718-3

[42] Caffarelli E Fatica A Prislei S De Gregorio E Fragapane P Bozzoni I. Processing of the intron-encoded U16 and U18 snoRNAs: the conserved C and D boxes control both the processing reaction and the stability of the mature snoRNA. *EMBO J* 1996;15 (5):1121–1131. 8605882 PMC450010

[43] Baserga SJ Yang XD Steitz JA. An intact Box C sequence in the U3 snRNA is required for binding of fibrillarin, the protein common to the major family of nucleolar snRNPs. *EMBO J* 1991;9 (10):2645–2651. 10.1002/j.1460-2075.1991.tb07807.x PMC452965 1714385

[44] Baxter-Roshek JL Petrov AN Dinman JD. Optimization of ribosome structure and function by rRNA base modification. *PLoS One* 2007;2 (1):e174. 17245450 10.1371/journal.pone.0000174 PMC1766470

[45] Basu A Das P Chaudhuri S Requirement of rRNA methylation for 80S ribosome assembly on a cohort of cellular internal ribosome entry sites. *Mol Cell Biol* 2011;31 (22):4482. 21930789 10.1128/MCB.05804-11 PMC3209261

[46] Ni J Tien AL Fournier MJ. Small nucleolar RNAs direct site-specific synthesis of pseudouridine in ribosomal RNA. *Cell* 1997;89 (4):565–573. 9160748 10.1016/s0092-8674(00)80238-x

[47] Meroueh M Grohar PJ Qiu J Santalucia J Jr Scaringe SA Chow CS. Unique structural and stabilizing roles for the individual pseudouridine residues in the 1920 region of *Escherichia coli* 23S rRNA. *Nucleic Acids Res* 2000;28 (10):2075–2083. 10773075 10.1093/nar/28.10.2075 PMC105375

[48] Ofengand J. Ribosomal RNA pseudouridines and pseudouridine synthases. *FEBS Lett* 2002;514 (1):17–25. 11904174 10.1016/s0014-5793(02)02305-0

[49] Lane BG Ofengand J Gray MW. Pseudouridine in the large-subunit (23 S-like) ribosomal RNA the site of peptidyl transfer in the ribosome? *FEBS Lett* 1992;302 (1):1–4. 1587345 10.1016/0014-5793(92)80269-m

[50] King TH Liu B McCully RR Fournier MJ. Ribosome structure and activity are altered in cells lacking snoRNPs that form pseudouridines in the peptidyl transferase center. *Mol Cell* 2003;11 (2):425–435. 12620230 10.1016/s1097-2765(03)00040-6

[51] Darzacq X Jády BE Verheggen C Kiss AM Bertrand E Kiss T. Cajal body-specific small nuclear RNAs: a novel class of 2′-O-methylation and pseudouridylation guide RNAs. *EMBO J* 2002;21 (11):2746–2756. 12032087 10.1093/emboj/21.11.2746 PMC126017

[52] Will CL Lührmann R. Spliceosomal UsnRNP biogenesis, structure and function. *Curr Opin Cell Biol* 2001;13 (3):290–301. 11343899 10.1016/s0955-0674(00)00211-8

[53] Kishore S Stamm S. The snoRNA HBII-52 regulates alternative splicing of the serotonin receptor 2C. *Science* 2006;311 (5758):230–232. 16357227 10.1126/science.1118265

[54] Kishore S Khanna A Zhang Z The snoRNA MBII-52 (SNORD 115) is processed into smaller RNAs and regulates alternative splicing. *Hum Mol Genet* 2010;19 (7):1153–1164. 20053671 10.1093/hmg/ddp585 PMC2838533

[55] Lykke-Andersen S Ardal BK Hollensen AK Damgaard CK Jensen TH. Box C/D snoRNP autoregulation by a cis-acting snoRNA in the NOP56 Pre-mRNA. *Mol Cell* 2018;72 (1):99–111e5. 30220559 10.1016/j.molcel.2018.08.017

[56] Scott MS Ono M Yamada K Endo A Barton GJ Lamond AI. Human box C/D snoRNA processing conservation across multiple cell types. *Nucleic Acids Res* 2012;40 (8):3676–3688. 22199253 10.1093/nar/gkr1233 PMC3333852

[57] Kim DS Camacho CV Nagari A Malladi VS Challa S Kraus WL. Activation of PARP-1 by snoRNAs controls ribosome biogenesis and cell growth via the RNA helicase DDX21. *Mol Cell* 2019;75 (6):1270–1285e14. 31351877 10.1016/j.molcel.2019.06.020 PMC6754283

[58] Schubert T Pusch MC Diermeier S Df31 protein and snoRNAs maintain accessible higher-order structures of chromatin. *Mol Cell* 2012;48 (3):434–444. 23022379 10.1016/j.molcel.2012.08.021

[59] Zhang Z Pi J Zou D microRNA arm-imbalance in part from complementary targets mediated decay promotes gastric cancer progression. *Nat Commun* 2019;10 (1):4397. 31562301 10.1038/s41467-019-12292-5 PMC6764945

[60] Sharma S Yang J van Nues R Specialized box C/D snoRNPs act as antisense guides to target RNA base acetylation. *PLoS Genet* 2017;13 (5):e1006804. 28542199 10.1371/journal.pgen.1006804 PMC5464676

[61] Bispo JA Pinheiro PS Kobetz EK. Epidemiology and etiology of leukemia and lymphoma. *Cold Spring Harb Perspect Med* 2020;10 (6):a034819. 31727680 10.1101/cshperspect.a034819 PMC7263093

[62] Wu J Xiao Y Sun J A single-cell survey of cellular hierarchy in acute myeloid leukemia. *J Hematol Oncol* 2020;13 (1):128. 32977829 10.1186/s13045-020-00941-y PMC7517826

[63] Döhner H Weisdorf DJ Bloomfield CD. Acute myeloid leukemia. *N Engl J Med* 2015;373 (12):1136–1152. 26376137 10.1056/NEJMra1406184

[64] Stadtfeld M Apostolou E Ferrari F Ascorbic acid prevents loss of Dlk1-Dio3 imprinting and facilitates generation of all-iPS cell mice from terminally differentiated B cells. *Nat Genet* 2012;44 (4):398–405. 22387999 10.1038/ng.1110 PMC3538378

[65] Herter EK Stauch M Gallant M Wolf E Raabe T Gallant P. snoRNAs are a novel class of biologically relevant Myc targets. *BMC Biol* 2015;13 (1):25. 25888729 10.1186/s12915-015-0132-6 PMC4430873

[66] Li T Zhou X Wang X Zhu D Zhang Y. Identification and characterization of human snoRNA core promoters. *Genomics* 2010;96 (1):50–56. 20353816 10.1016/j.ygeno.2010.03.010

[67] Vendramini E Giordan M Giarin E High expression of miR-125b-2 and SNORD116 noncoding RNA clusters characterize ERG-related B cell precursor acute lymphoblastic leukemia. *Oncotarget* 2017;8 (26):42398–42413. 28415578 10.18632/oncotarget.16392 PMC5522075

[68] Ronchetti D Mosca L Cutrona G Small nucleolar RNAs as new biomarkers in chronic lymphocytic leukemia. *BMC Med Genomics* 2013;6 (1):27. 24004562 10.1186/1755-8794-6-27 PMC3766210

[69] Dupuis-Sandoval F Poirier M Scott MS. The emerging landscape of small nucleolar RNAs in cell biology. *Wiley Interdiscip Rev RNA* 2015;6 (4):381–397. 25879954 10.1002/wrna.1284 PMC4696412

[70] Mourtada-Maarabouni M Hedge VL Kirkham L Farzaneh F Williams GT. Growth arrest in human T-cells is controlled by the non-coding RNA growth-arrest-specific transcript 5 (GAS5). *J Cell Sci* 2008;121 (Pt 7):939–946. 18354083 10.1242/jcs.024646

[71] Mourtada-Maarabouni M Pickard MR Hedge VL Farzaneh F Williams GT. GAS5, a non-protein-coding RNA, controls apoptosis and is downregulated in breast cancer. *Oncogene* 2009;28 (2):195–208. 18836484 10.1038/onc.2008.373

[72] Coccia EM Cicala C Charlesworth A Regulation and expression of a growth arrest-specific gene (gas5) during growth, differentiation, and development. *Mol Cell Biol* 1992;12 (8):3514–3521. 1630459 10.1128/mcb.12.8.3514-3521.1992 PMC364604

[73] Nakamura Y Takahashi N Kakegawa E The GAS5 (growth arrest-specific transcript 5) gene fuses to BCL6 as a result of t(1;3)(q25;q27) in a patient with B-cell lymphoma. *Cancer Genet Cytogenet* 2008;182 (2):144–149. 18406879 10.1016/j.cancergencyto.2008.01.013

[74] Amson R Pece S Marine J-C Fiore PPD Telerman A. TPT1/TCTP-regulated pathways in phenotypic reprogramming. *Trends Cell Biol* 2013;23 (1):37–46. 23122550 10.1016/j.tcb.2012.10.002

[75] Berquet L Valleron W Grgurevic S Small nucleolar RNA expression profiles refine the prognostic impact of IGHV mutational status on treatment-free survival in chronic lymphocytic leukaemia. *Br J Haematol* 2016;172 (5):819–823. 26095450 10.1111/bjh.13544

[76] Tanaka R Satoh H Moriyama M Intronic U50 small-nucleolar-RNA (snoRNA) host gene of no protein-coding potential is mapped at the chromosome breakpoint t(3;6)(q27;q15) of human B-cell lymphoma. *Genes Cells* 2000;5 (4):277–287. 10792466 10.1046/j.1365-2443.2000.00325.x

[77] Dong XY Guo P Boyd J Implication of snoRNA U50 in human breast cancer. *J Genet Genomics* 2009;36 (8):447–454. 19683667 10.1016/S1673-8527(08)60134-4 PMC2854654

[78] Dong XY Rodriguez C Guo P SnoRNA U50 is a candidate tumor-suppressor gene at 6q14.3 with a mutation associated with clinically significant prostate cancer. *Hum Mol Genet* 2008;17 (7):1031–1042. 18202102 10.1093/hmg/ddm375 PMC2923223

[79] Mahajan N Wu HJ Bennett RL Sabotaging of the oxidative stress response by an oncogenic noncoding RNA. *FASEB J* 2017;31 (2):482–490. 28148777 10.1096/fj.201600654R PMC5240659

[80] Oliveira V Mahajan N Bates ML The snoRNA target of t(4;14) in multiple myeloma regulates ribosome biogenesis. *FASEB Bioadv* 2019;1 (7):404–414. 32095781 10.1096/fba.2018-00075 PMC6996358

[81] Ronchetti D Todoerti K Tuana G The expression pattern of small nucleolar and small Cajal body-specific RNAs characterizes distinct molecular subtypes of multiple myeloma. *Blood Cancer J* 2012;2 (11):e96. 23178508 10.1038/bcj.2012.41 PMC3511933

[82] Mourtada-Maarabouni M Hasan AM Farzaneh F Williams GT. Inhibition of human T-cell proliferation by mammalian target of rapamycin (mTOR) antagonists requires noncoding RNA growth-arrest-specific transcript 5 (GAS5). *Mol Pharmacol* 2010;78 (1):19–28. 20421347 10.1124/mol.110.064055 PMC2912054

[83] Yoshihama M Nakao A Kenmochi N. snOPY: a small nucleolar RNA orthological gene database. *BMC Res Notes* 2013;6:426. 24148649 10.1186/1756-0500-6-426 PMC4015994

[84] Xie J Zhang M Zhou T Hua X Tang L Wu W. Sno/scaRNAbase: a curated database for small nucleolar RNAs and cajal body-specific RNAs. *Nucleic Acids Res* 2007;35 (Database issue):D183–D187. 17099227 10.1093/nar/gkl873 PMC1669756

[85] Yang JH Zhang XC Huang ZP snoSeeker: an advanced computational package for screening of guide and orphan snoRNA genes in the human genome. *Nucleic Acids Res* 2006;34 (18):5112–5123. 16990247 10.1093/nar/gkl672 PMC1636440

[86] Schwartz S Bernstein DA Mumbach MR Transcriptome-wide mapping reveals widespread dynamic-regulated pseudouridylation of ncRNA and mRNA. *Cell* 2014;159 (1):148–162. 25219674 10.1016/j.cell.2014.08.028 PMC4180118

[87] Dai Q Moshitch-Moshkovitz S Han D Nm-seq maps 2′-O-methylation sites in human mRNA with base precision. *Nat Methods* 2017;14 (7):695–698. 28504680 10.1038/nmeth.4294 PMC5712428

[88] Li X Fu XD. Chromatin-associated RNAs as facilitators of functional genomic interactions. *Nat Rev Genet* 2019;20 (9):503–519. 31160792 10.1038/s41576-019-0135-1 PMC7684979

[89] Wang WT Chen TQ Zeng ZC The lncRNA LAMP5-AS1 drives leukemia cell stemness by directly modulating DOT1L methyltransferase activity in MLL leukemia. *J Hematol Oncol* 2020;13 (1):78. 32552847 10.1186/s13045-020-00909-y PMC7302350

[90] Croce CM Calin GA. miRNAs, cancer, and stem cell division. *Cell* 2005;122 (1):6–7. 16009126 10.1016/j.cell.2005.06.036

